# A Modeling Framework to Frame a Biological Invasion: *Impatiens glandulifera* in North America

**DOI:** 10.3390/plants12071433

**Published:** 2023-03-24

**Authors:** Oğuzhan Kanmaz, Tuğçe Şenel, H. Nüzhet Dalfes

**Affiliations:** Department of Climate and Marine Sciences, Eurasia Institute of Earth Sciences, Istanbul Technical University, Maslak, 34469 Istanbul, Turkey

**Keywords:** invasive species, *Impatiens glandulifera*, North America, hybrid modeling, species distribution modeling, agent-based modeling, MaxEnt, invasion modeling

## Abstract

Biological invasions are a major component of global environmental change with severe ecological and economic consequences. Since eradicating biological invaders is costly and even futile in many cases, predicting the areas under risk to take preventive measures is crucial. *Impatiens glandulifera* is a very aggressive and prolific invasive species and has been expanding its invasive range all across the Northern hemisphere, primarily in Europe. Although it is currently spread in the east and west of North America (in Canada and USA), studies on its fate under climate change are quite limited compared to the vast literature in Europe. Hybrid models, which integrate multiple modeling approaches, are promising tools for making projections to identify the areas under invasion risk. We developed a hybrid and spatially explicit framework by utilizing MaxEnt, one of the most preferred species distribution modeling (SDM) methods, and we developed an agent-based model (ABM) with the statistical language R. We projected the *I. glandulifera* invasion in North America, for the 2020–2050 period, under the RCP 4.5 scenario. Our results showed a predominant northward progression of the invasive range alongside an aggressive expansion in both currently invaded areas and interior regions. Our projections will provide valuable insights for risk assessment before the potentially irreversible outcomes emerge, considering the severity of the current state of the invasion in Europe.

## 1. Introduction

Global environmental change is an ongoing and anthropogenic issue involving various components [1]. Biological invasions, together with climate change, are the leading drivers [2,3] of severe ecological consequences such as species extinctions, which are irreversible, and biodiversity loss, which takes millions of years to return to the former levels known from the periods following big extinction events, according to fossil records [4,5]. An invasive species causes, or is likely to cause, ecological and economic consequences alongside harm to human and animal well-being [6,7,8]. Ecosystem impacts caused by biological invasions, such as altering the function and structure of ecosystems [9], homogenization of biotas [10,11], and changing disturbance regimes [12,13,14], are diverse in severity and sometimes idiosyncratic [15]. Economic costs are also tremendous [16] and are expected to rise [17]. The impacts on ecosystem services [18], and especially threats to food security, constitute an important source of concern [19].

Today, invasive species introductions continue to increase without any sign of saturation worldwide [20]. In the face of this ever-growing problem, the most important lessons learned from the long history of biological invasions can be summarized as: the eradication of an established biological invader is virtually impossible [21], prevention is the most effective strategy [22], and the first step of prevention is to identify high-risk areas [23]. Consequently, model projections for potential range expansions play a crucial role in the prevention and controlling of biological invasions, since they can serve as early warning systems [24].

Over the last decades, owing to the increasing computational power and availability of biogeographic [25] and environmental data [26,27], correlative species distribution models (SDMs) have become widely popular due to their ability to produce credible, defensible, and repeatable information for conservation, management, and risk assessment [28,29,30]. Biological invasions have been a major application field of SDMs to be utilized for making two types of projections: the determination of the areas under invasion risk and prediction of the possible outcomes of an invasion under environmental change [31,32] via their spatial and temporal transferability [33]. Despite a vast number of studies involving different SDM methods, some of which with strategies to improve the results [34,35,36,37], the applicability of SDMs on biological invasions has been a source of controversy due to the potential violation of the basic assumptions of equilibrium, niche conservatism, and lack of dispersal limitation [38]. Hybrid modeling, the integration of distinct modeling approaches to represent complex, integrated systems [39,40], stands out as an alternative for modeling biological invasions to overcome the inherent limitations of SDMs [31,41,42] and incorporate the processes and interactions that SDMs cannot address to make more reliable projections with various promising examples [43,44,45,46]. Agent-based models (ABMs), which simulate populations or systems of populations as being composed of discrete agents [47] and have been used in many biological invasion studies [48,49,50,51], are a useful potential component for such hybrid models. In this regard, a hybrid model, which consists of SDM and ABM, can be highly useful in simulating biological invasions. While SDM provides suitability layers to be utilized by ABM, ABM simulates the essential processes such as dispersal, establishment, and biotic relations.

*Impatiens glandulifera* is native to the Western Himalayas and is considered one of the most prolific invasive species across the Northern Hemisphere [52]. It was introduced to Europe in the first half of the 19th century as an ornamental garden plant and for its high-quality nectar to be used in beekeeping [53,54]. While its primary habitats in the native range are forests and forest gaps [55], it primarily invades riparian habitats and only in recent decades has it been observed to colonize forests [56]. As rivers and water streams are its main dispersal vectors, anthropogenic means and animals are also known contributors to its seed dispersal [57].

Although *I. glandulifera* is defined as a transformer species [58], conclusions about its impact on species composition, diversity, and richness vary from “insignificant” [59,60,61] to “prominent” [62,63,64,65,66]. However, the differences can be attributed to factors such as the initial conditions of the invasion sites [67], the residence time in the invasion site [68], and local species composition [62]. Among other problems, *I. glandulifera* may cause erosion [69] due to its extremely shallow root system [70], increased eutrophication risk as a result of erosion [58], and problems related to stream management [53,59].

The invasive range of *I. glandulifera* currently extends across the Northern Hemisphere due to the combined effects of factors such as its wide environmental tolerance, the release from coevolved natural enemies [71,72], and opportunities caused by anthropogenic-land-use change [56]. Its invasive range in the Southern Hemisphere somewhat mirrors the northern pattern by the equator. *I. glandulifera* occurrence is currently recorded in more than 40 countries and registered as invasive in 30 countries [73].

Currently, the invasive range of *I. glandulifera* in North America is mainly concentrated in the Great Lakes, New England, and Canadian Maritimes regions in the East, Pacific Northwest, and British Columbia, along with Alaska and California as latitudinal extremes in the West. Occurrences are reported in at least 15 states in the U.S.A. and all provinces of Canada [73,74,75,76,77], and the species is considered “naturalized” in several of them [70,78,79]. The earliest records in North America date back to 1883 in Norwich, Connecticut [80]; 1901 in Ottawa, Ontario [70]; and 1912 in Port Huron, Michigan [81] in the East and 1937 in Burnaby, British Columbia [70]; and 1944 in Washington State [79] in the West. Its first occurrence in Alaska, where it is considered naturalized, was as recent as 2004 [82]. Despite its known presence in Mexico, occurrence records are not available through online databases [75,83]. Yet, for North America, literature on *I. glandulifera* is quite limited ([70,80] are some examples) compared to the vast number of studies for Europe (e.g., [53,66,84,85,86,87,88]). Consequently, there is still much to be understood about the fate of *I. glandulifera* in North America under climate change, considering the history and severity of the current state of the invasion in Europe.

We developed a hybrid and spatially explicit framework that consists of: (i) a correlative component (CC) utilizing species distribution modeling and (ii) an agent-based component (ABC) using agent-based modeling methods. These components are highly interconnected and work in a loop. In this loop, ABC is responsible for the generation, dispersal, and constraining of the establishment of agents in a dynamic heterogeneous environment, and CC uses these agents as occurrences to produce bioclimatic suitability layers. The occurrence of an invasion is only possible with the proper combination of the conditions in the recipient region [89], which is coined with the concept of invasion windows [90]. Additionally, the invasion law of minimum states that the least favorable one can be the determinant that makes the timing of introduction crucial alongside the place of introduction in a dynamic environment, since the invasion’s success is dependent on multiple factors [91,92]. Accordingly, while the framework proceeds in yearly time steps, the established success of agents relies on the invasion windows that are simulated via the environmental data layers. For the implementation presented here, we constructed species-specific procedures for ABC and utilized MaxEnt, one of the most preferred SDM methods [93], in the CC. This study aimed to project the *I. glandulifera* invasion in North America for the period 2020–2050 under the RCP 4.5 scenario and provide continental scale projections for an overview of the potential range expansion. Our projections will be one of the few studies conducted on *I. glandulifera* in North America, and thus will be a valuable contribution to the literature in addition to providing synoptic insights for preventive management, control, and conservation plans.

## 2. Materials and Methods

The framework was developed with the statistical language R (R version; 3.6.1, [94]) in the RStudio development environment (RStudio version; 1.2.1335, [95]). The MaxEnt algorithm was incorporated into the framework with the MaxEnt software for modeling species niches and distributions [96] via the Dismo package in R [97].

### 2.1. Occurrence Data

**Figure 1:**
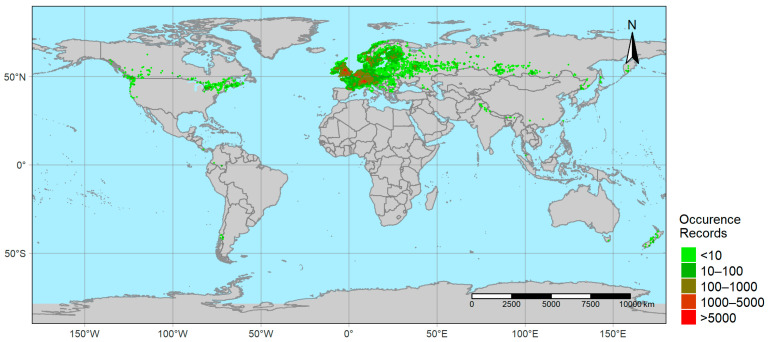
Occurrence records of *Impatiens glandulifera* (based on GBIF data).

Occurrence records of *I. glandulifera* were obtained from the website of the Global Biodiversity Information Facility (GBIF) [98]. The data were cleaned by excluding the duplicate records and records without georeferencing and was filtered to contain only the observational data on the “basis of record” criteria (recorded as “human observation” in the basisOfRecord column). With this step, more than 300,000 occurrence records globally and 1522 from the region of interest, North America (respectively Occ_Global_ and Occ_NA_ hereafter), were obtained (Figure 1). While Occ_NA_ was used as the input for the simulations for correlative model training and initial agent generation, Occ_Global_ was utilized during the determination of the values of ABC variables.

### 2.2. Environmental Data Layers

Environmental layers can be categorized into three types: static, yearly, and emergent. Static environmental layers contain the data, which are assumed to be unchanged over the study period, such as elevation, slope, and soil pH. The yearly environmental data layers were pre-calculated for the years in the simulation period to be used in the corresponding time steps (e.g., climatic and land use data layers). Lastly, the emergent layers, unlike the static and yearly layers, were generated by the framework during the simulations, such as occurrence maps and climatic suitability maps.

#### 2.2.1. Climatic Data Layers

The raw climatic dataset constitutes the basis of all climatic layers used in the model. It contains daily maximum temperature, minimum temperature, and precipitation for the period between 1990 and 2050, with a spatial resolution of 0.25°.

The raw climate dataset was constructed from two data sets to cover historical data and projections. The historical part was the ERA5 climate reanalysis data set [99] of the European Centre for Medium-Range Weather Forecasts (ECMWF), obtained from the Copernicus Climate Change Service Data Store [100]. The projections part was the downscaled projections under the RCP 4.5 scenario of MIROC5 General Circulation Model (GCM) [101], included in The NASA Earth Exchange Global Daily Downscaled Projections (NEX-GDDP) dataset [102,103]. Linear scaling, a common method to minimize the bias between GCM outputs and observed data, was applied to the projections to make these two data sets compatible [104,105]. Yearly bioclimatic variable (BCY) data layers, long-term bioclimatic variable (BCL) data layers, and accumulated chilling hours (ACHs) data layers were derived from the raw climatic data set.

BCY data layers were calculated from temporally upscaled (from daily to monthly) climatic raw data for the period of 1990–2050 via the Dismo package in R [97]. Then, the 30-year means were calculated from BCYs for the period 2020–2050 to obtain long-term bioclimatic variable data layers, and each layer was named with the last year of the period (e.g., BCL_2020_ is the mean of BCYs between 1991 to 2020).

Bioclimatic variables were selected based on the permutation importance, a measure that depended on the final MaxEnt model instead of the path to obtain the model itself, and it determined the contribution of each variable by permuting the values of the variables among the presence and background training points by measuring the decrease in the training area under the curve (AUC) [106]. This operation was conducted via the ENMeval package of R [107]. The block method was selected for spatial partitioning because of its known merits in cases involving temporal and spatial transfer [107].

Based on the mean permutation importance for each bioclimatic variable, which was calculated from the results of 50 repetitions conducted with Occ_NA_ and BCL_2020_, eight bioclimatic variables with a sum of 87.57% permutation importance were determined to narrow down the BCY and BCL: BIO13 (Precipitation of Wettest Month), BIO11 (Mean Temperature of Coldest Quarter), BIO15 (Precipitation Seasonality), BIO1 (Annual Mean Temperature), BIO9 (Mean Temperature of Driest Quarter), BIO6 (Min Temperature of Coldest Month), BIO18 (Precipitation of Warmest Quarter), and BIO4 (Temperature Seasonality). Permutation importance percentages of predictors are given in the Supplementary Materials, Table S1.

The ACHs in the February–March period (from day-of-year 32 to 90) were calculated for the period of 2005–2020 to construct the ACH data layers with a base temperature of 5 °C. Since chill hours calculations require hourly data, the temporal resolution of raw daily climatic data was temporally downscaled (from daily to hourly) via the chillR package in R [108].

#### 2.2.2. Land Use Data Layers

Land use layers were derived from The Land-Use Harmonization 2 (LUH2) datasets for RCP 4.5 scenarios, with a spatial resolution of 0.25° [109,110]. Yearly layers were composited with the five classes of agricultural projections, including C3 annual crops, C3 nitrogen-fixing crops, C3 perennial crops, C4 annual crops, and C4 perennial crops.

#### 2.2.3. Elevation and Slope Data Layers

The Median Statistic product with a 15 arc seconds resolution, from Global Multi-resolution Terrain Elevation Data 2010 (GMTED2010), developed by The U.S. Geological Survey (USGS) and the National Geospatial-Intelligence Agency (NGA), was selected as the elevation layer and downloaded from the Earth Resources Observation and Science (EROS) Center [111,112]. The slope layer was derived from the elevation layer via the terrain function of the raster package in R [113].

#### 2.2.4. Soil pH Data Layers

Soil pH data at a depth of 0.15 m with a 7.5 arc seconds resolution was obtained from SoilGrids—a data set generated at the International Soil Reference and Information Centre (ISRIC)—to be utilized as the soil pH layer [114,115].

### 2.3. Structure of the Framework

**Figure 2:**
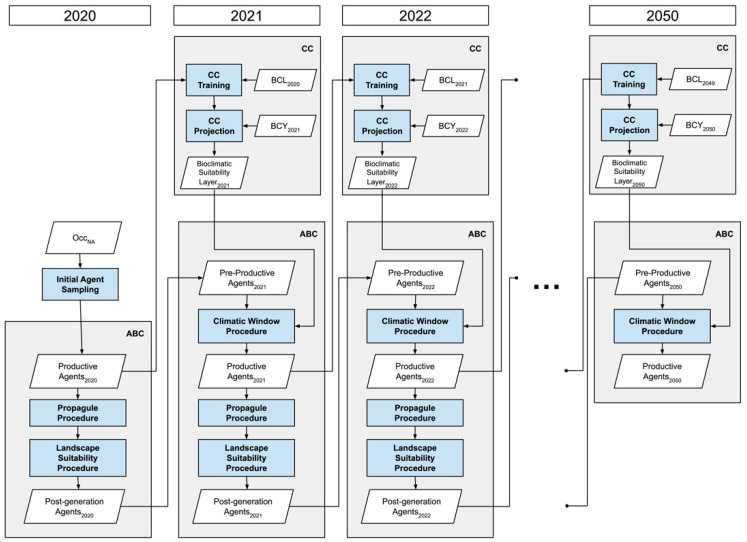
Workflow of the framework throughout the simulation period 2020–2050. (ABC: Agent-Based Component; CC: Correlative Component; Occ_NA_: Initial Occurrence Records in North America; BCL: long term bioclimatic variables; BCT: Yearly bioclimatic variables).

As aforementioned, the framework consists of an ABC and a CC. The ABC generates and evaluates the agent populations to be utilized as occurrence records for model training by the CC. The CC makes projections for the ABC to be utilized as bioclimatic suitability layers. The processes of both components operate in a loop that spans consecutive time steps during the simulations (Figure 2). In a hierarchical sense, the CC primarily works at the population level by making projections based on the productive agent populations, while the ABC operates at the individual level by generating and processing agents.

#### 2.3.1. Correlative Component

The CC performs model training with the long-term climatic conditions, which gradually change throughout the simulation period, and the latest occurrence is recorded to make projections for the upcoming year. Accordingly, BCL and the productive agent population of the previous time step are used to train a MaxEnt model as predictors and occurrence records, respectively. Then, the trained MaxEnt model is transferred to the BCY of the current time step to make projections in the form of bioclimatic suitability (BCS) layers, which the ABC will utilize.

#### 2.3.2. Agent Based Component

**Figure 3:**
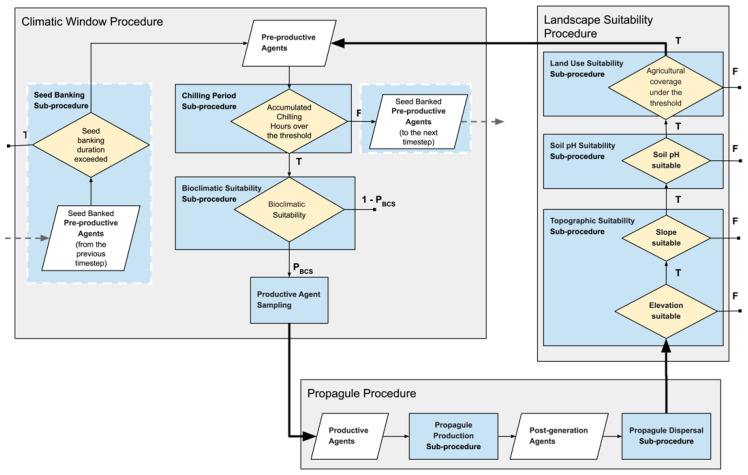
Workflow of the Agent-Based Component in a time step (P_BCS_: Bioclimatic Suitability of a cell).

The ABC has three procedures, the Climatic Window Procedure, Propagule Procedure, and Landscape Suitability Procedure, in order of execution in a time step, and operates with three types of agents: productive agents, post-generation agents, and pre-productive agents. The workflow of the ABC is given in Figure 3. Productive agents represent the mature units with the capacity for seed production and correspond to the occurrence records. The initial productive agents are sampled from occurrence data with the assumption that all occurrence records were captured in their mature form. Post-generation agents are the generated and dispersed seeds produced by mature agents to germinate in the next time step. Pre-productive agents consist of ungerminated seeds and seed-banked pre-productive agents from previous time steps.

##### Climatic Window Procedure

The first phase of the ABC, in order of execution in a time step, is the Pre-productive Phase, which consists of: the chilling period, seed banking, bioclimatic suitability, and productive agent sampling sub-procedures. All environmental layers in this phase are yearly environmental layers.

*I. glandulifera* needs a chilling period in order to break dormancy and germinate [116], which occurs between February and March period [117]. Despite the required duration and temperature for proceeding of germination, reported as longer than 45 days at 4 °C [118] and one month at 5 °C [119], it is known that the parameters are highly variable depending on the seed properties and chilling period consistency. The chilling period was added to the framework with the following approach.

To determine a threshold based on the ACHs in the February–March period, 1234 occurrence records between 2005 to 2020 were assessed with the ACH layers of the respective years, with thresholds ranging between 600 to 1080 chilling hours. The threshold was determined as 720 chilling hours (30 days), since ~96% of the occurrences were observed to be recorded in the cells with ACH values over this threshold, which does not push the suitable zone unrealistically northward or southward. Occurrence records in the cells below the threshold may result from local habitat conditions that could not be captured by the coarse resolution of the ACH layers or other factors that are known to affect the germination process [120]. The chilling period sub-procedure uses the ACH layer of the corresponding year in accordance with the threshold value to assess the pre-productive agents. If the ACH value of the cell is above the threshold, the pre-productive agent proceeds to the Bioclimatic Suitability sub-procedure. If not, the agent is evaluated by seed the banking sub-procedure.

*I. glandulifera* is not known to form persistent and long-lasting seed banks [121], yet the seeds can survive longer than a year [86]. Accordingly, the seed banking duration variable, which determines in how many time steps a banked pre-productive agent is kept to be evaluated in the case of failure to pass the chilling period sub-procedure, was set as 1 year. The first seed banking is performed in the second time step, and the first evaluation of a seed-banked pre-productive agent occurs in the third time step, since the first time step starts with the propagule procedure.

The BCS layer of the corresponding time step, which the CC generates, is utilized in Bioclimatic Suitability sub-procedure. Although these layers can be thresholded to transform the probability/suitability data to presence/absence data with various methods [122], they were kept “as is” in the simulations. The cell values were used as probabilities to take advantage of the continuous and probabilistic nature of the projections to capture the gradients instead of sharp boundaries [123].

The pre-productive agents that can pass the sub-procedures of the Climatic Window Procedure are considered productive agents and correspond to the simulated occurrence records to be utilized by the CC. The Productive Agent Sampling sub-procedure involves a grid sampling operation with a 0.25° resolution occurrence map and is conducted in accordance with the sample per cell variable, which was set to 1 per cell for the simulations to identify the productive agents to proceed to the Propagule Procedure. While the grid sampling operation of the first step is performed with the initial occurrence records to reduce the inherent sampling bias in the occurrence data [45], which can lead to bias towards more intensively surveyed cells [124], it is responsible for scaling the population processed by the framework by determining the maximum number of productive agents on a cell in the occurrence map. In this regard, it must be noted that the population densities in the cells do not represent real populations due to the applied scaling.

##### Propagule Procedure

The second procedure of the ABC is the Propagule Procedure. It is mainly responsible for simulating the propagule pressure via its sub-procedures: Propagule Production and Propagule Dispersal. Propagule Procedure is the only procedure that does not use environmental layers due to its relatively generic structure.

The propagule Production sub-procedure performs the production of post-generation agents. The seed production of *I. glandulifera* is a well-documented subject both qualitatively and quantitatively [125,126,127]. However, determining the number of propagules produced by productive agents is not a straightforward task, as scaling is a common challenge of ABM applications. Thus, calibration simulations were conducted for different values of the propagule count to observe the model’s behavior. Consequently, 50 was determined as the propagule count, since further increments did not show drastic changes on the projected final invasive range and its latitudinal extremes, despite becoming computationally demanding with considerably longer runtimes.

Determination of dispersal direction and distance for the post-generation agents is performed in the Propagule Dispersal sub-procedure. For this implementation, an unsophisticated method was followed to simulate the short and mid-distance dispersal, and dispersal vectors were not explicitly distinguished. A random dispersal direction, drawn from the uniform distribution, was assigned to each post-generation agent; the dispersal distance for each post-generation agent was determined via a truncated negative exponential distribution. Maximum and mean dispersal distances were set as 38 km and 5 km, respectively [117], and the latter was used to determine the rate parameter of the negative exponential distribution.

##### Landscape Suitability Procedure

The third procedure of the ABC is the Landscape Suitability Procedure, which contains the Topographic Suitability, Soil pH Suitability, and Land Use Suitability sub-procedures. Since the successful transformation from post-generation agents to pre-productive agents is only possible if all the conditions are met, the execution order of the sub-procedures is not important.

The topographic Suitability sub-procedure evaluates the post-generation agents by using the elevation and slope layers. The elevational distribution of *I. glandulifera* differs between its native and invaded ranges. While it can grow on elevations exceeding 4000 m in its native range [55,117,128], it mostly occurs on lower elevations up to 600 m [59,125,126,127] in the invaded ranges. *I. glandulifera* mostly occurs on flat or slightly sloped terrains, up to 40° from horizontal, yet it is also known to be recorded on steeply sloping banks exceeding 40° [55]. The analysis conducted based on the Occ_Global_ and Occ_NA_ with elevation and slope data layers showed that 98.3% and 99.9% of the occurrences were under 1000 m elevation, respectively, and 99.9% of the occurrences were recorded on terrains with less than 20° median slope. Thus, the simulations were realized with a 1000 m maximum elevation and a 20° maximum slope limit. While the determined maximum slope limit was lower than that of the reports in the literature, the difference can be attributed to the coarse resolution of the slope data.

The Soil pH Suitability sub-procedure assesses the post-generation agents by using the soil pH data layer in accordance with the known soil pH tolerance of *I. glandulifera,* which is between 4.5 and 7.7 [129]. If the value of the cell is above or below the tolerance interval, the establishment is inhibited. Soil pH at 15 cm depth was selected, based on the shallow root length of *I. glandulifera,* which is about 10–20 cm [70,130].

The Land Use Suitability sub-procedure evaluates the post-generation agents by using the land use data layer of the corresponding time step and inhibits the establishment on the cells containing agricultural land over the threshold value. *I. glandulifera* is not known to infest agricultural lands, yet it is observed to occur on the field margins in its native range [55,128]. The same pattern has also been reported in its invaded range [70,131]. The analysis, conducted based on the Occ_Global_ and Occ_NA_ with 2020 land use data layers, showed that all the occurrence records were distributed over the cells with less than 80% agricultural coverage, except for one record. Consequently, 80% was determined as the maximum agricultural coverage limit for the simulations.

### 2.4. Initialization

All 50 simulations conducted for the study began with the grid sampling of the occurrence data from Occ_NA_ to determine the initial agents, which are the productive agents of the first time step. These agents were kept throughout the simulations to be processed by the ABC and CC, alongside the generated agents. Thus, the first time step did not include the first two sub-procedures of the Climatic Window Procedure and proceeded to the Propagule Procedure after the grid-sampling was performed by the Agent Sampling sub-procedure. Accordingly, every simulation was initialized with an equal number of invaded cells. However, due to the stochasticity, which resulted from sampling, the initial distribution of the agents was slightly different for each simulation. All defined variables and the assigned values used in the simulations are given in the Supplementary Materials, Table S2.

The initial extent of the simulations was determined based on the minimum bounding rectangle of the initial agent’s coordinates. Minimum and maximum longitude and latitudes were rounded with floor and ceiling functions. Then, a margin of 2° was added to obtain the initial extent. Following the first time step, the yearly extents were determined dynamically with the productive agent coordinates in the corresponding previous time step with the same method. The geographic extents were utilized for cropping the environmental layers and background point sampling.

### 2.5. Output

**Figure 4:**
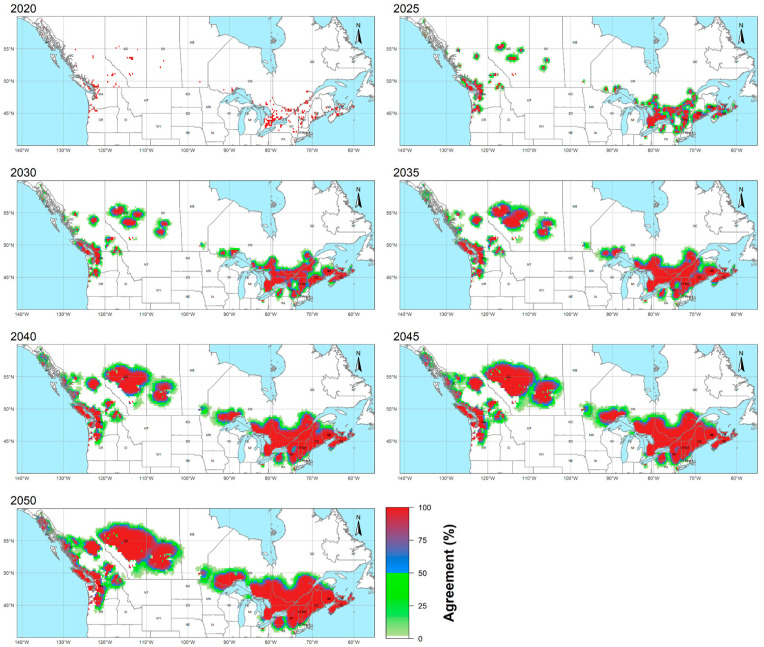
5-year inter-simulation agreement maps for the projected invasive range of *I. glandulifera* in North America.

The yearly and 5-year inter-simulation agreement maps were constructed based on productive agents in the corresponding period to show the percentage of the simulations predicting the invasion of a particular cell. While the 5-year agreement maps were utilized for analysis to avoid the yearly fluctuation (Figure 4), the yearly agreement maps were presented as an animation, given in the Supplementary Materials (see Supplementary Animation).

The invaded cells or cell cumulations, which were initially present or formed during the simulations via merging and isolated from the others, are described with a loose term “focus” (plural foci) in the analysis. This naming is due to their role as the propagule source while expanding to the suitable areas. State, province, or geographic region and direction were used to signify the spatial context where they occur/were found (e.g., Ohio focus, Intermountain Region foci).

The foci, which the framework could not process throughout the simulations, were described as “irresponsive”. By the exclusion of the irresponsive California (3 cells), Newfoundland and Labrador (1 cell), and Northwest Territories (1 cell) foci, the analysis’ extent was determined as 140° W–60° W and 40° N–60° N. Due to reasons such as the somewhat independent invasion histories and the differences observed during the analysis, the extent was separated as east (60° W to 100° W) and west (100° W to 140° W) halves. All analyses were conducted based on moderate-agreement (>50%) cells.

Alongside the simulations, One-factor-at-a-time (OFAT) sensitivity analysis was also conducted. The OFAT sensitivity analysis method, which is performed by changing one parameter from a selected base parameter set (nominal set), while all other parameters are fixed to their nominal values, is used to determine the relationship between the varied parameter and output. OFAT provides an understanding of model mechanisms by demonstrating if the response is linear or nonlinear of the tipping points and whether there are tipping points where drastic responses occur with small parameter changes [132]. For the analysis, six parameters of ABC (Mean Dispersal Distance, Maximum Dispersal Distance, Propagule Production, Maximum Agricultural Coverage, Accumulated Chilling Hours, and Maximum Elevation) were used for five values with 10 simulations per case to detect the relationship between the variables and the invaded cell counts in the final 5-year period (Supplementary Table S3). The plots were constructed from the results, and the agreement maps for the final 5-year period of each case are given in the Supplementary Figures S1–S4.

## 3. Results

### 3.1. Geographic Overview

In the eastern half of the analysis’ extent, the projected invasive range was observed to cover provinces of the Maritimes (New Brunswick, Prince Edward Island, Nova Scotia), the northern states of the New England region (Vermont, New Hampshire, and Maine), the majority of New York, and the southern parts of Ontario and Quebec, with a high agreement. The southern boundary was observed to cross the northern parts of southern New England (Connecticut, Rhode Island, and Massachusetts), and Pennsylvania and was formed by the expansion of the neighboring areas. The Ohio and New Jersey foci, both located on the southern border of the invasive range in the eastern half, were observed to be stagnant and isolated from the rest of the high agreement zone of the invasive range. The northern boundary was formed by the northward expansion of the Quebec and Ontario foci, which were initially scattered on the shores of the Saint Lawrence River and Great Lakes, respectively.

The projected invasive range on the western half was primarily determined by the Pacific mountain ranges and was, consequently, more fragmented in comparison to the eastern half. The narrow range on the shores of British Columbia and Washington, which was limited by coastal ranges, was observed to continue to the north of Oregon, following the Cascades. No expansion was projected on the shores starting from the south of the Olympic Peninsula. In the Intermountain region of British Columbia, an expansion of several distinct foci, which were between the Rockies and coastal ranges, was observed. While the Fraser Plateau’s focus was the most prominent of these foci, the southernmost focus was slightly expanded to the north of Oregon, Idaho, and Montana. In Alaska, although the spread on the shores was limited, the Alexander Archipelago, especially the Admiralty, Baranof, and Chichagof Islands, was observed to be severely impacted. Arguably, the most striking projection obtained from the simulations was the formation that originated from the scattered foci of Alberta and Saskatchewan. The southern boundary was observed to be an arch crossing the north of Oregon, the southernmost focus of the Intermountain region, and south of Alberta–Saskatchewan. The northern boundary was determined primarily by Alaska and the north of Alberta–Saskatchewan.

### 3.2. Longitudinal and Latitudinal Gradients

For detecting the changes in longitudinal and latitudinal gradients over the simulation period, results were also assessed regarding their Invaded Cell Count (ICC), the number of the invaded cells over each of the five-year periods (with the expectation of 2020, which was showing the initially invaded cell count), and Interperiod Invaded Cell Increment (IPI) difference between ICC values of consecutive periods, within longitudinal bands of 10° and latitudinal bands of 5°.

**Figure 5:**
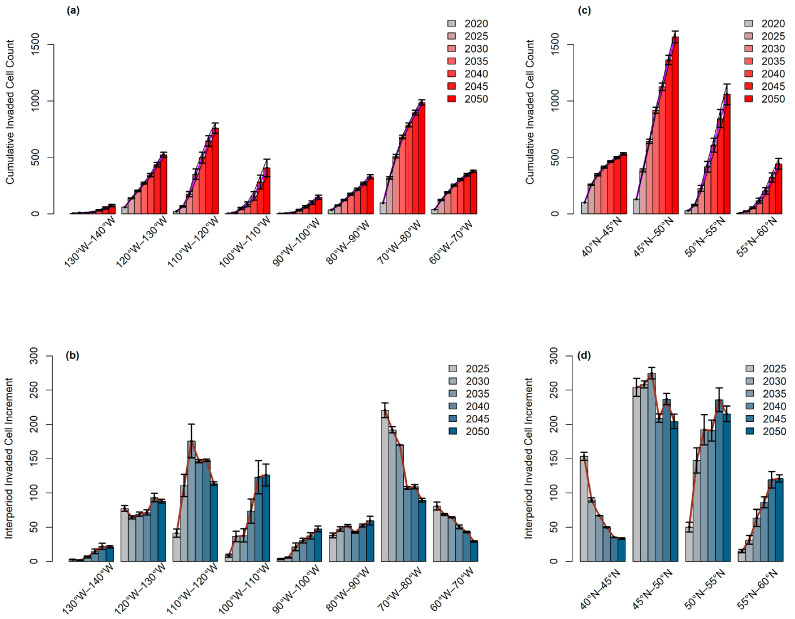
Invaded Cell Counts (ICC) and Interperiod Invaded Cell Increment (IPI) of 10° Longitudinal and 5° latitudinal bands for 5-year periods. (**a**) ICC of 10° longitudinal bands, (**b**) IPI of 10° longitudinal bands, (**c**) ICC of 5° latitudinal bands, (**d**) IPI of 5° latitudinal bands.

As seen in Figure 5a,b, 60° W–70° W and 70° W–80° W longitudinal bands, located in the easternmost of the invasive range and containing the East Coasts of Canada and the U.S.A., were the only bands to show an IPI decrease, which led to a prominent slowing trend in ICC throughout the simulation period. The 110° W–120° W band, which had the second highest final ICC, following the 60° W–70° W band, and primarily contained Alberta, was observed to show a slowing trend in ICC after mid-simulation in accordance with decreasing IPI. The 100° W–110° W band, which mainly contains Saskatchewan, did not show any significant slowing trend in ICC or IPI decrease. The linear ICC increase, due to IPI without a significant trend throughout the simulation period, was observed in the bands: 80° W–90° W, which contains the northern shores of the Great Lakes, and 120° W–130° W, which contains the west shores and the Intermountain region. In 90° W–100° W, containing Alaska, and 130° W–140° W, which contains Lake Superior, ICC was observed to increase after a lag period, albeit being relatively weak.

As for the latitudinal bands (see Figure 5c,d), the southernmost band, 40° N–45° N, demonstrated the most significant IPI decrease and ICC-slowing trend among all bands, including the longitudinal bands. IPI was also observed to have a negative trend for the 45° N–50° N band, which had the highest final ICC, despite being far less significant. For the 50° N–55° N and 55° N–60° N bands, primarily due to the increase in the Canadian Prairies, an accelerating ICC was observed, while IPI showed a slight decrease at the end of the period.

### 3.3. Latitudinal Shifts

**Figure 6:**
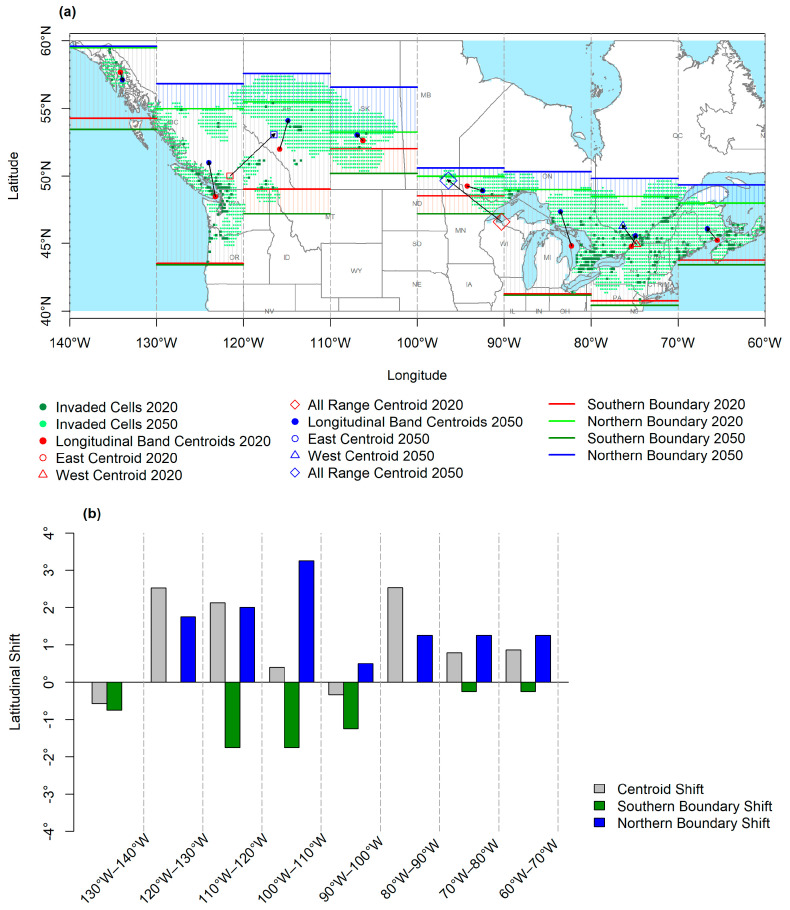
Shifts of centroids and boundaries for 10° longitudinal bands. (**a**) Initial (2020) and final (2050) centroids and northern/southern boundaries. The black arrows show the shift direction of the centroids. (**b**) Zero-centered shifts of centroids and southern/northern boundaries. Positive values of the y axis represent northward, negative values of the y axis represent southward shifts.

The extent of the range shift was determined by evaluating the centroids and latitudinal extremes of the initial (2020) and final 5-year period (2050) distributions. Centroids were calculated with a rather unsophisticated method by calculating the mean values of latitudes and longitudes of invaded cells (see Figure 6a,b). Through the simulation period, the centroid of all invaded cells in the entire range was shifted 3.04° northward, while the shift for the eastern half was 1.35° and the west was 3.05°. Similarly, latitudes of the northern and southern extremes of each band were determined for the initial and final period distributions. Then, their respective differences were calculated to evaluate the shifts in the latitudinal boundaries. The mean shift of the northern boundary was observed to be 1.4° for the entire range, 1.06° for the eastern half, and 1.75° for the western half. The mean shift of the southern boundary was observed to be 0.75° for the entire range, 0.43° for the eastern half, and 1.06° for the western half.

When the latitudinal bands were examined individually, progression of the northern boundary, except the 140° W–130° W band, was observed to be common and pronounced compared to the progression of the southern boundary, which was primarily more pronounced for the bands initially containing foci on the higher latitudes. Individual centroids of the bands paralleled the progression of the northward boundaries, and all centroids were observed to shift northward, except the 140° W–130° W and 90° W–100° W bands.

### 3.4. Predictive Performance of Correlative Component

**Figure 7:**
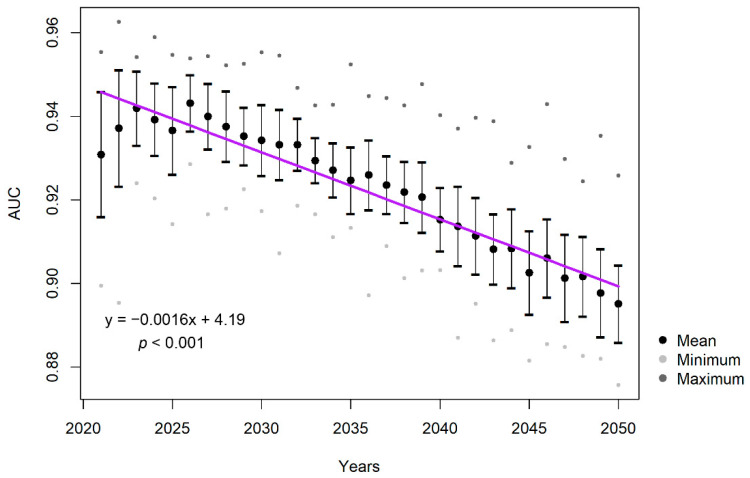
The area under the curve (AUC) values of MaxEnt models over the simulation period 2021–2050.

The predictive performance of the CC, MaxEnt, was evaluated with AUC. AUC is one of the most extensively used statistics, measuring the ability of a model to discriminate the sites in which species are present from which the species are absent. AUC score ranges between 0 and 1, while 1 shows perfect discrimination and 0.5 implies the discrimination is not different from any random guess [133]. The mean AUC values of all simulations were observed to decrease through the simulation period with a significant trend (*p* < 0.01). However, considering the mean AUC for the first and last time step were 0.93 and 0.89, respectively, that decrease is acceptable for the time frame of the simulations (see Figure 7).

## 4. Discussion

The results from the simulations showed that *I. glandulifera* would increase its occurrence in the regions where it was currently present/reported in both the east and west parts, alongside an aggressive expansion in the interior regions in North America under the RCP 4.5 scenario. In particular, Alberta and Saskatchewan in the Canadian Prairies were projected to be a major invasive range, despite the currently limited *I. glandulifera* distribution. Many studies reported wide-scale poleward shift patterns due to climate change for a wide range of species (e.g., [134,135]). Invasive plants are not an exception (e.g., [136,137]), as this pattern was also projected for various invasive plants (e.g., [138,139,140,141]. Beerling [142] projected a northward invasive range expansion for *I. glandulifera* in Europe under climate change (solely the minimum winter temperature and growing degree days were used as predictors) [87], and these projections were supported by the increasing occurrence reports from higher latitudes in Europe in recent decades [73]. Following that, Tabak and von Wettberg [80] stated that the emergence of a similar pattern, i.e., a northward expansion, may also be expected for North America. Considering these, the northward expansion pattern shown by our results corresponded with the predictions in the reviewed literature.

The northern border of the projected invasive range was observed as roughly following the deciduous-boreal forest ecotone [143] in the East and the prairie-forest biome border [144] in the Alberta–Saskatchewan region in the West. The rapid warming in the boreal forests (approximately twice as fast as the global average [145]) and the projected northward shifts of warmer climate zones [146], e.g., the climatic shifts from prairies to the boreal forests [147,148], are expected to cause significant disturbances that can affect individual species and ecosystems and can lead to biome-level changes [149]. Partial or widespread removal of resident communities due to these disturbances [150], e.g., the formation of treefall gaps in ecotones, can increase the establishment and recruitment success of northward migrating temperate species [151]. Since *I. glandulifera* commonly occurs in ecotones [57], such conditions can increase invasion success. *I. glandulifera* seedlings are known to establish quickly in disturbed woodland sites [120], including woodland clearings and sparse woodlands [60] and, under some circumstances, to suppress woodland regeneration by facilitating the establishment of other species [58,152].

Drought stress, which has been more severe and frequent due to climate change, can affect *I. glandulifera* negatively by surpassing its physiological resistance strategies [153]. This finding is also supported by Beerling’s [142] predictions on the relationship between the southern boundary of *I. glandulifera* and summer droughts and the limited distribution of *I. glandulifera* in the south of Europe [56,73,87]. Consequently, the limited southward expansion of the projected invasive range and its geographical pattern is in accordance with the predicted increase in the drought severity and frequency with climate change for the USA and Canada [154,155,156,157].

*I. glandulifera* was recorded in Europe in the mountainous regions [66,88] in some cases at elevations exceeding 1000 m [53,158]. Additionally, recent studies reported an *I. glandulifera* spread in forests up to the timberline [60]. Moreover, elevational shifts of suitable habitats for tree populations due to climate change were also predicted [159]. Considering these facts, the projected expansion of invasive range in the mountainous regions in the west could be more severe by reaching elevations higher than the 1000 m elevation limit in our simulations. Another potential risk in recent decades is the increased impact of bark beetles with direct and indirect effects of climate change in the region [160], as the disturbances caused by bark beetle outbreaks facilitate a faster spread of *I. glandulifera* in forests [56,161].

The dispersal mechanism implemented in our framework simulated short and mid-distance dispersal, which led to the formation of patterns representing the spreading of invasive species with diffusion-like processes in short distances [162]. On the other hand, long-distance dispersal is typically connected to anthropogenic activities [163], and determining the potential consequences of these activities would constitute a limitation for the framework. *I. glandulifera* was observed to spread with forest machinery, river gravel, and topsoil, which is used for construction projects [56,164], ship ballasts [70], etc. Roads and railways are also known as its dispersal vectors [165,166]. However, trade is the most crucial factor in the spread of invasive species [167], and, as an ornamental, this is very true for *I. glandulifera.* The rise in the trading of invasive plants via e-commerce [168] in recent decades without effective biosecurity measures [169] constitutes a serious problem in many cases. It may serve the spread of invasive plants in an unpredictable and uncontrolled fashion. Given that the introduction of *I. glandulifera* outside of its native range was for horticultural purposes [117] and is still considered a popular ornamental with a remarkable economic value [170], anthropogenic impacts can drastically change the course of the invasion we projected.

To some extent, all SDMs are affected by the quality and completeness of, or biases in, the data [171,172]. Our study is not an exception and may be affected by such bias. Wallacean shortfalls [173], the incompleteness of species distribution data, and spatial and temporal biases are inherent in occurrence data [174,175]. Importantly, in the case of invasive species, lacking information such as whether the occurrences are recorded from an early stage of invasion [176] or whether they still reflect a stable relationship with the environment [177], constitute a source of potential problems in the context of equilibrium, which leads to bold yet unavoidable assumptions. Despite its crucial role as the largest initiative that provides occurrence data [178], GBIF is known to have pronounced biases [179]. As an example of the mentioned incompleteness, the reported presence of *I. glandulifera* in Anchorage, Alaska [82] was not included in the simulations, which may constitute a potential source of underestimation in the corresponding region, since it was absent in the data obtained from GBIF.

While our projections can be considered satisfactory as a preliminary attempt to provide an invasive range outline on a continental scale for North America, the framework, in its current state, does not include every factor that affects *I. glandulifera* invasion. The inclusion of these factors, e.g., biotic interactions, which can improve the accuracy of results, is planned for the further steps of our study. In consideration with the high competitive abilities of I. glandulifera, primarily due to allelopathic effects on co-occurring plant species, including dominant ones [180], soil fungal and bacterial communities [63], and even aquatic species in riparian habitats [181], are striking examples that emphasize the importance of such interactions on the invasion process. Although our approach is mainly centered around constraining the establishment and dispersal, in accordance with the modular structure, the framework can be extended to simulate the processes facilitating establishment. Additionally, implementing a more sophisticated dispersal mechanism to simulate the dispersal vectors (such as animals [57,117]) explicitly, especially the inclusion of river networks alongside higher resolution environmental data in finer geographic scales, can be highly beneficial to make more accurate projections. It is also worth mentioning that the approach we follow can be adapted to be utilized for other species after the generalization of the design, which we plan for the upcoming steps of the study, regardless of the current ad hoc structure of the framework.

Eradication of *I. glandulifera* is costly [182] and even futile after its spread in interconnected lowland watercourses [52]. Eradication efforts can lead to an invasional meltdown, as the sites after the removal are much more prone to be invaded by other invasive plants [62]. Thus, habitat restoration is a must, along with removal [58]. Chemical control is an available [75] but a not yet viable choice for riparian habitats [183]. Mechanical control is expensive and laborious [184]. The effects of fungal pathogens on biological control vary between the populations [185]. *I. glandulifera*, as Bieberich suggests [186], is a back-seat driver facilitated by previous ecosystem changes and also a driver of further changes. Thus, habitat stability is more important than the habitat type to explain its impacts [58]. In this respect, management and conservation strategies should be evaluated for local conditions. Preventive measures and monitoring are needed to keep the current distribution under control and prevent further spread to habitats that are already under the impact of global environmental change.

## Data Availability

All data used in this study can be reached via the links provided in the references.

## Supplementary Materials

The following supporting information can be downloaded at: https://www.mdpi.com/article/10.3390/plants12071433/s1, Figure S1: Results of the OFAT analysis for the evaluated variables; Figure S2: Inter-simulation agreement maps of the projected invasive ranges Maximum Dispersal Distance and Mean Dispersal Distance simulations; Figure S3: Inter-simulation agreement maps of the projected invasive ranges Propagule Production and Accumulated Chilling Hours simulations; Figure S4: Inter-simulation agreement maps of the projected invasive ranges Maximum Agricultural Coverage and Maximum Elevation simulations; Table S1: Permutation importance percentages of the bioclimatic variables used in the simulations as predictors for MaxEnt model training; Table S2: Assigned values of the defined variables in Agent Based Component; Table S3: The parameter sets of One-factor-at-a-time (OFAT) sensitivity analysis; Animation S1: The supplementary animation which was constructed based on the yearly intersimulation agreement maps.
