# Supplementary material for: A Modeling Framework to Frame a Biological Invasion: Impatiens glandulifera in North America
Source: Plants (Basel). 2023 Mar 24;12(7):1433. doi: 10.3390/plants12071433 (PMC10097319; doi:10.3390/plants12071433)

Table S1. Permutation importance percentages of the bioclimatic variables used in the simulations as predictors for MaxEnt model training

	Bioclimatic Variable	Permutation Importance (%)
BIO13	Precipitation of Wettest Month	22.55
BIO11	Mean Temperature of Coldest Quarter	20.15
BIO15	Precipitation Seasonality	15.04
BIO1	Annual Mean Temperature	12.96
BIO9	Mean Temperature of Driest Quarter	7.26
BIO6	Min Temperature of Coldest Month	4.05
BIO18	Precipitation of Warmest Quarter	3.22
BIO4	Temperature Seasonality	2.34
		**Sum: 87.57**

Table S2. Assigned values of the defined variables in Agent Based Component

Procedure	Sub-procedure	Variable	Value	Unit
Climatic Window	Chilling period	Accumulated Chilling Hours	720	hour
Climatic Window	Seed Banking	Seed Banking Duration	1	year
Climatic Window	Productive Agent Sampling	Occurence Sample	1	per cell
Propagule	Propagule Production	Propagule Count	50	per agent
Propagule	Propagule Dispersal	Mean Dispersal Distance	5	km
Propagule	Propagule Dispersal	Maximum Dispersal Distance	38	km
Landscape Suitability	Topographic Suitability	Maximum Elevation	1000	m
Landscape Suitability	Topographic Suitability	Maximum Slope	20	degrees
Landscape Suitability	Soil pH Suitability	Minimum suitable soil Ph	4.5	
Landscape Suitability	Soil pH Suitability	Maximum suitable soil Ph	7.7	
Landscape Suitability	Land Use Suitability	Maximum Agricultural Coverage	80	%

Table S3. The parameter sets of One-factor-at-a-time (OFAT) sensitivity analysis

	Abbreviation	Mean Dispersal Distance (km)	Maximum Dispersal Distance (km)	Propagule Production (propagule)	Accumulated Chilling Hours (h)	Maximum Agricultural Coverage (%)	Maximum Elevation (m)	Conducted Simulations
**Nominal Set**	NS	5	38	50	720	80	1000	50
**Mean Dispersal Distance Simulations**	$MnDD_{2.5}$	**2.5**	38	50	720	80	1000	10
	$MnDD_{3.75}$	**3.75**	38	50	720	80	1000	10
	$MnDD_{6.25}$	**6.25**	38	50	720	80	1000	10
	$MnDD_{7.5}$	**7.5**	38	50	720	80	1000	10
**Maximum Dispersal Distance Simulations**	$MxDD_{18}$	5	**18**	50	720	80	1000	10
	$MxDD_{28}$	5	**28**	50	720	80	1000	10
	$MxDD_{48}$	5	**48**	50	720	80	1000	10
	$MxDD_{58}$	5	**58**	50	720	80	1000	10
**Propagule Production Simulations**	PP_{30}	5	38	**30**	720	80	1000	10
	PP_{40}	5	38	**40**	720	80	1000	10
	PP_{60}	5	38	**60**	720	80	1000	10
	PP_{70}	5	38	**70**	720	80	1000	10
**Accumulated Chilling Hours Simulations**	ACH_{600}	5	38	50	**600**	80	1000	10
	ACH_{840}	5	38	50	**840**	80	1000	10
	ACH_{960}	5	38	50	**960**	80	1000	10
	ACH_{1080}	5	38	50	**1080**	80	1000	10
**Maximum Agricultural Coverage Simulations**	MEC_{50}	5	38	50	720	**50**	1000	10
	MEC_{60}	5	38	50	720	**60**	1000	10
	MEC_{70}	5	38	50	720	**70**	1000	10
	MEC_{90}	5	38	50	720	**90**	1000	10
**Maximum Elevation Simulations**	ME_{700}	5	38	50	720	80	**700**	10
	ME_{800}	5	38	50	720	80	**800**	10
	ME_{900}	5	38	50	720	80	**900**	10
	ME_{1100}	5	38	50	720	80	**1100**	10

Figure S1. Results of the OFAT analysis for the evaluated variables. (a) Maximum Dispersal Distance (b) Mean Dispersal Distance (c) Propagule Production (d) Accumulated Chilling Hours (e) Maximum Agricultural Coverage (f) Maximum Elevation

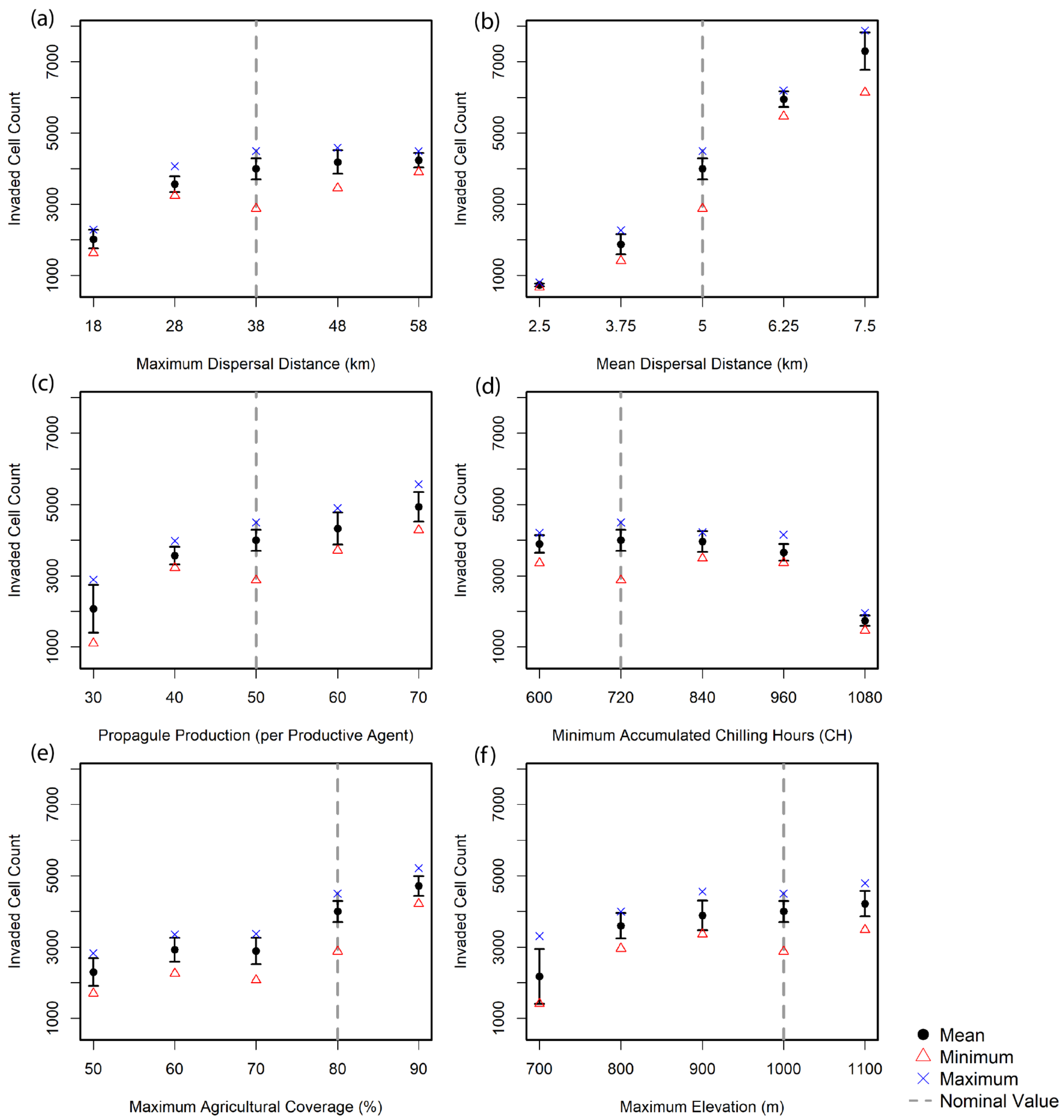

Figure S2. Inter-simulation agreement maps of the projected invasive ranges Maximum Dispersal Distance (a to e) and Mean Dispersal Distance (f to j) simulations. The abbreviations are given in the Supplementary Table-3. (a)$MxDD_{2.5}$, (b)$MxDD_{3.75}$, (c)NS, (d)$MxDD_{6.25}$, (e)$MxDD_{7.5}$ (f)$MnDD_{18}$, (g)$MnDD_{28}$, (h)NS, (i)$MnDD_{48}$, (j)$MnDD_{58}$

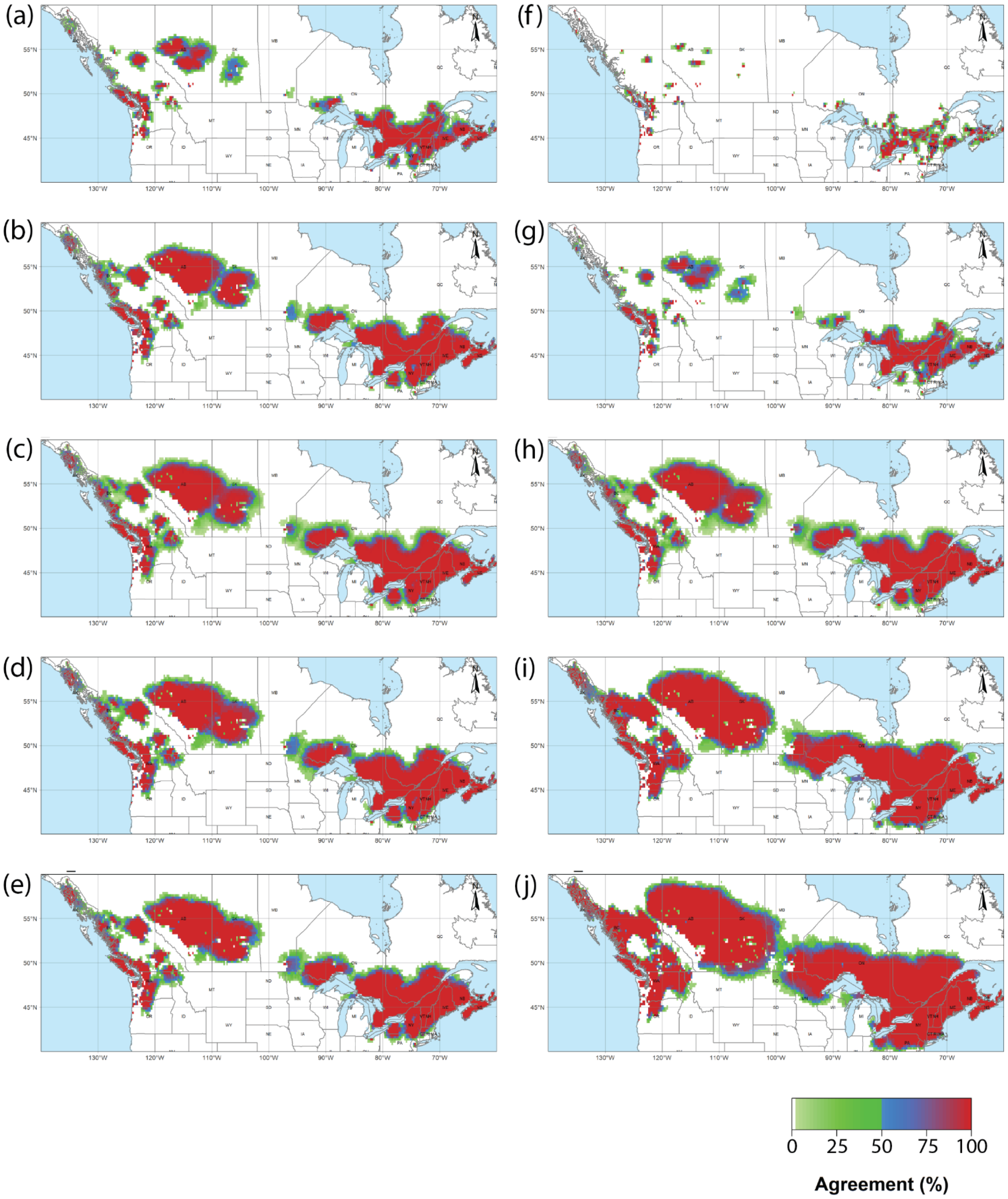

Figure S3. Inter-simulation agreement maps of the projected invasive ranges Propagule Production (a to e) and Accumulated Chilling Hours (f to j) simulations. The abbreviations are given in the Supplementary Table-3. (a)PP_{30}, (b)PP_{40}, (c)NS, (d)PP_{60}, (e)PP_{70}, (f)ACH_{600}, (g)NS, (h)ACH_{840}, (i)ACH_{960},(j)ACH_{1080}

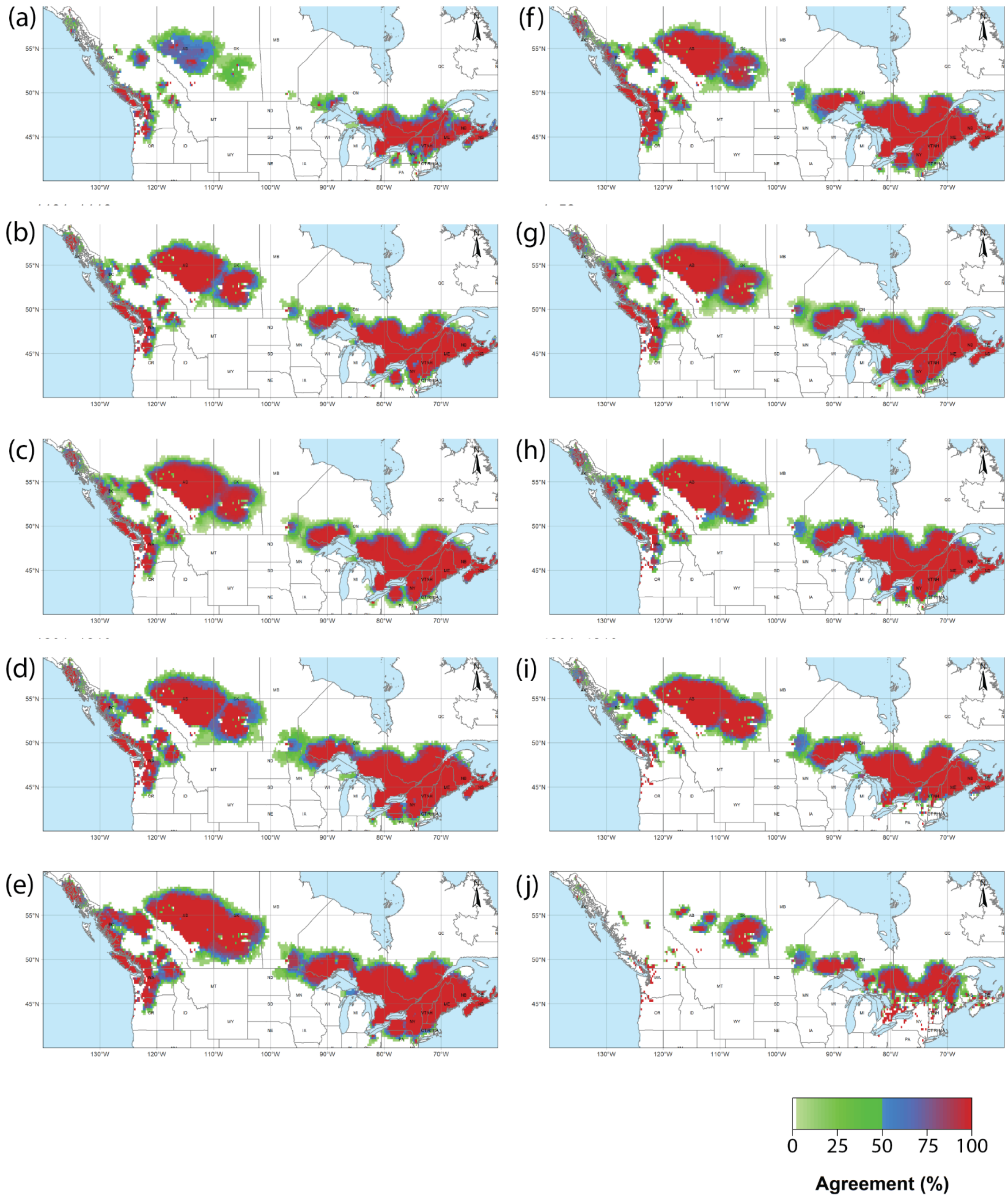

Figure S4. Inter-simulation agreement maps of the projected invasive ranges Maximum Agricultural Coverage (a to e) and Maximum Elevation (f to j) simulations. The abbreviations are given in the Supplementary Table-3. (a)MEC_{50} , (b)MEC_{60} , (c)MEC_{70} , (d)NS, (e)MEC_{90}, (f)ME_{700} , (g)ME_{800} , (h)ME_{900} , (i)NS, (j)ME_{1100}

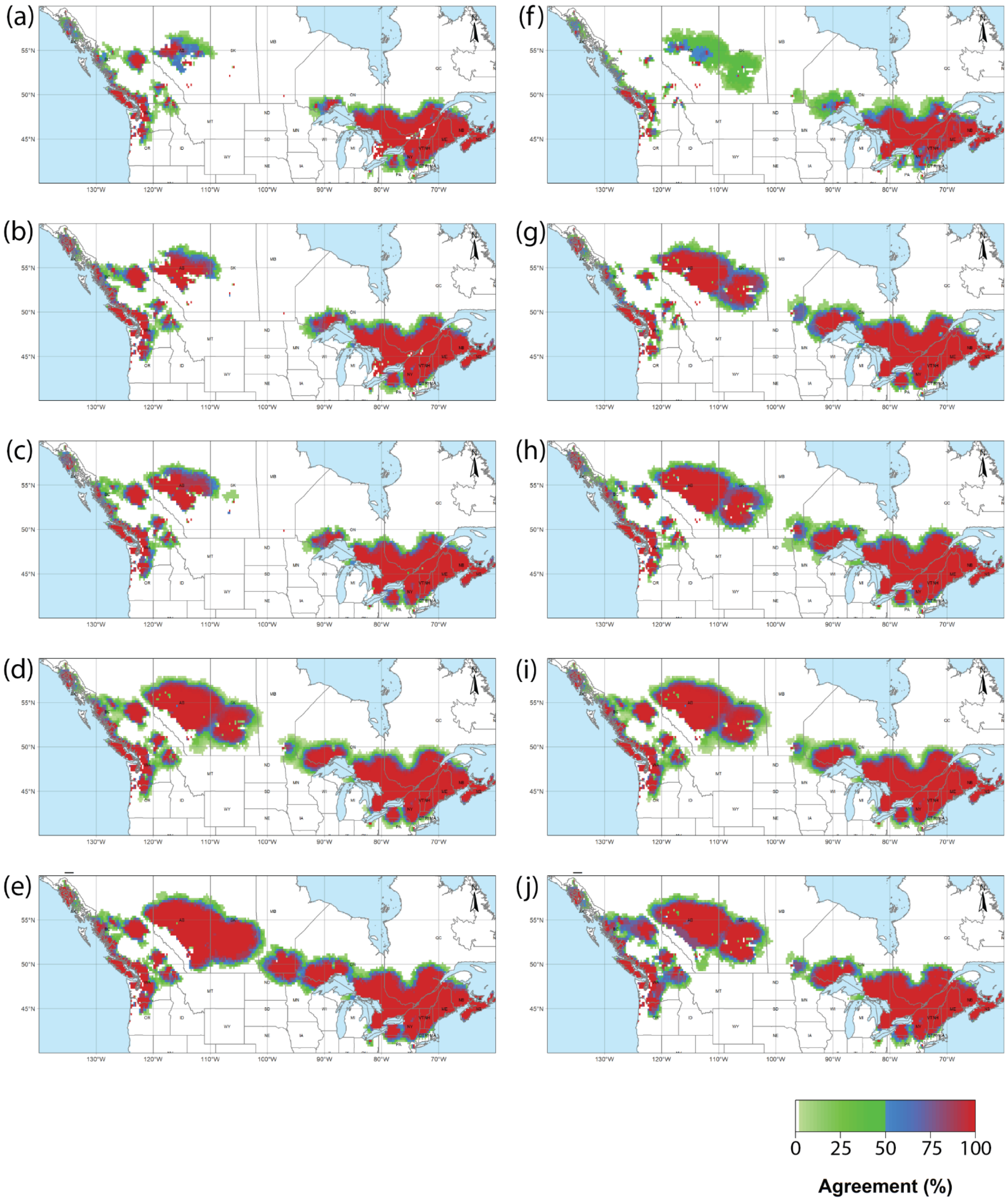

Supplement: Supplementary file 1 [file plants-12-01433-s001.zip › Supplementary Material_KO_ŞT_HND .pdf]
