# Supplementary figures and images for: A Modeling Framework to Frame a Biological Invasion: Impatiens glandulifera in North America
Source: Plants (Basel). 2023 Mar 24;12(7):1433. doi: 10.3390/plants12071433 (PMC10097319; doi:10.3390/plants12071433)

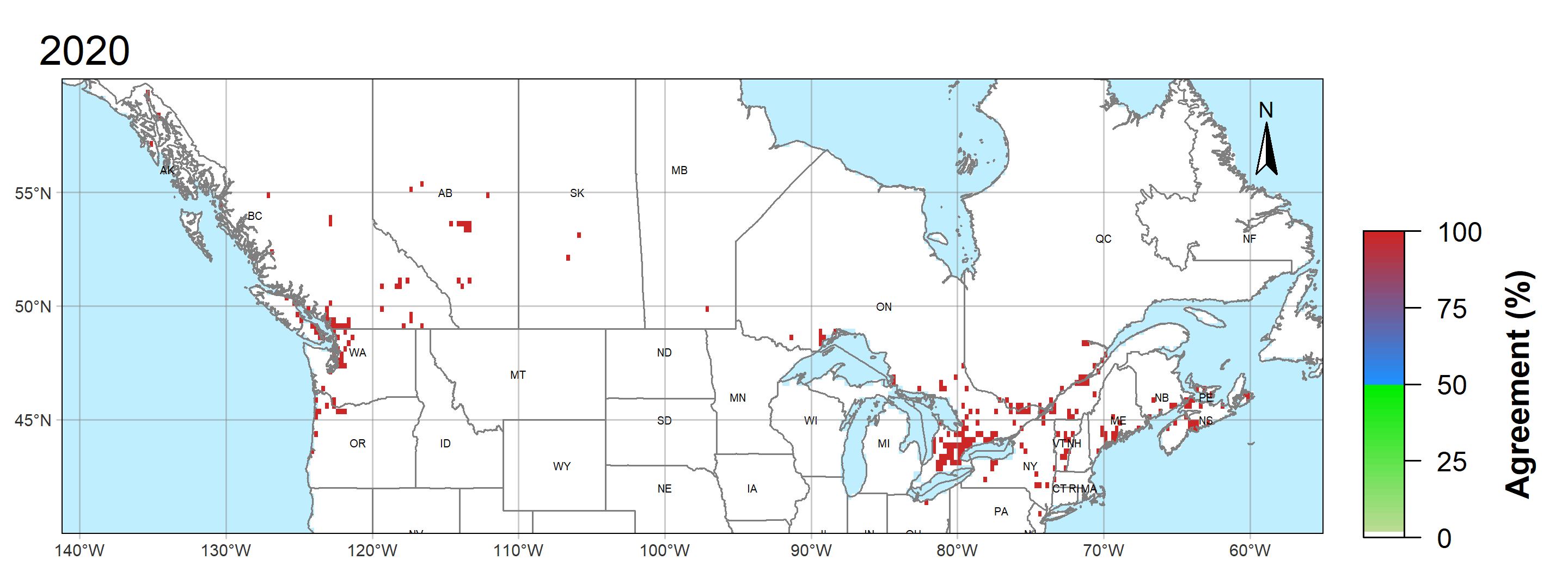

Supplement: Supplementary file 1 [file plants-12-01433-s001.zip › Suplementary Animation_KO_ŞT_HND.gif]
